# Effects of the COVID-19 Pandemic on Chinese Graduate Students’ Learning Activities: A Latent Class Analysis

**DOI:** 10.3389/fpsyg.2022.877106

**Published:** 2022-04-26

**Authors:** Jinqing Wang, Zhengyan Liang, Minqiang Zhang, Derong Kang, Qing Zeng

**Affiliations:** ^1^School of Psychology, South China Normal University, Guangzhou, China; ^2^Key Laboratory of Brain, Cognition and Education Sciences (South China Normal University), Ministry of Education, Guangzhou, China; ^3^Center for Studies of Psychological Application, South China Normal University, Guangzhou, China; ^4^Guangdong Key Laboratory of Mental Health and Cognitive Science, South China Normal University, Guangzhou, China

**Keywords:** COVID-19 pandemic, graduate students, learning activity, latent class analysis, mental health

## Abstract

To investigate the impact of the pandemic on graduate students’ learning activities, a series of questionnaires were distributed to graduate students in universities across China, and 2,818 responses were collected. A latent class analysis was performed to classify the effects of the pandemic on graduate students’ learning activities. Then, a multinomial logistic regression analysis and an analysis of variance analysis were carried out to explore the impact of demographic variables on the classification and their mental health status. The analysis identified four latent classes: “the overall less affected” (34.83%), “the overall more affected” (31.97%), “course activities were more affected” (19.40%), and “social activities were more affected” (13.79%). The multinomial logistic regression analysis indicated that during the pandemic, the learning activities of graduate students in all grades were affected to varying degrees, and the impacts on second-year and third-year graduate students were greater than those of first-year graduate students. The analysis of variance revealed that the scores for anxiety, depression, and social anxiety of “the overall more affected” were significantly greater than those of the other three groups, and nearly one-third of students belonged to this class, suggesting that more attention and care should be given to these students. As a result of the COVID-19 pandemic, a number of graduate students have suffered mental problems (anxiety and depression). Under the current backdrop of a new normal, schools and teachers should pay attention to graduate students’ mental health, providing targeted assistance to different types of students.

## Introduction

COVID-19 broke out at the end of 2019 and spread all over the world at an unprecedented speed. There was a total of over 400 million confirmed cases from the COVID-19 outbreak as of March 2022, with more than 200 countries and territories hit by the pandemic (Worldometers, 2021).

In China, to prevent the spread of the pandemic to campus and to ensure the safety and health of staff and students, a policy initiative called “suspending classes without stopping learning” was launched by the Ministry of Education (Ministry of Education of the Peoples Republic of China, 2020). All universities had implemented school closure, switching offline teaching to online education. However, the new form of online teaching and studying from home had disrupted the learning of students in higher education, and home quarantine had also exerted a considerable impact on their lives. Studies have shown that the impacts of the pandemic and lockdown on students in higher education are mainly reflected in three aspects:

First, changes in learning patterns. Offline learning was substituted by online learning. Some students found it challenging to adapt to the unfamiliar online learning environment (Blake et al., 2021; Farias Bezerra et al., 2021), and others lacked a stable network environment or a favorable learning environment at home; thus, their learning outcomes were also affected (Kapasia et al., 2020; Rasheed et al., 2020; Zhang et al., 2020).

Second, changes in social patterns. Students’ communications with teachers and classmates were reduced when studying from home (Blake et al., 2021). Due to the lockdown, it was difficult for students to have face-to-face contact with friends or relatives, nor could they meet and travel with their family and friends (Cao et al., 2020; Liu and He, 2020; Ma and Miller, 2020).

Third, student mental health was affected. Previous studies have shown that public health emergencies could have unfavorable psychological impacts on college students (Mei et al., 2011). During the pandemic, because of long-term isolation, reduced interpersonal interaction, and the lack of social support, students were susceptible to negative emotions such as fear, social isolation, anxiety, and depression (Cao et al., 2020; Liu and He, 2020; Ma and Miller, 2020).

As mentioned above, the pandemic outbreak has had a great impact on various aspects of college students’ lives, especially in learning and social patterns. Course and social activities are crucial parts in student life (Nguyen, 2017). In terms of course activities, the teaching model has shifted from offline to online, which leads to changes in both course arrangement and assessment arrangement (Barrot et al., 2021). Social support refers to perceived or actual help that an individual receives from the community, social network, relatives, and friends (Lin et al., 1986). Previous studies have shown that students’ academic performance is affected by the social support of parents, teachers, and peers (Plunkett and Bámaca-Gómez, 2003; Alfaro et al., 2006). When communicating with tutors and classmates, students can perceive and obtain actual assistance from them and perform better in their learning (Chen, 2005). However, in times of COVID-19, students experience majors changes in their learning environment, including curriculum teaching and social interaction, which leads to less learning engagement among students (Wang and Cai, 2021). In addition, online education is prone to problems such as unstable network environment, difficulty for students to engage and adapt, which worsens the learning outcomes (Kapasia et al., 2020; Rasheed et al., 2020; Blake et al., 2021). Regarding the mode of communication, online communication has caused great inconvenience to the interaction between students and their tutors (Jowsey et al., 2020; Blake et al., 2021). The above-mentioned factors have had a great impact on the learning activities of graduate students.

In addition, in the face of COVID-19, high levels of depression, anxiety, and stress were reported by students (Islam et al., 2020; Odriozola-González et al., 2020). According to the stress and coping theory developed by Lazarus and Folkman (1984), psychological stress is seen as a particular relationship between individuals and the environment that is perceived as exceeding their resources and jeopardizing their wellbeing. If individuals fail to cope with their negative emotions, their physical and mental health would be damaged under long-term stress, easily resulting in problems such as depression, anxiety, and posttraumatic stress disorder (Wang et al., 2020). There are a number of unfavorable influences during the pandemic that would lead to unpleasant psychological outcomes among graduate students. For example, research shows that the absence of social interaction can cause a higher level of anxiety (Cao et al., 2020). The transition of teaching models may result in poor academic performance for students, which raises their anxiety and depression levels (Basheti et al., 2021). Moreover, compared with other student groups, postgraduates’ arduous scientific research tasks result in higher mental stress (Zhu and Si, 2013) and more serious mental health problems (Zhao et al., 2004; Leal Filho et al., 2021). With COVID-19, the impacts on graduate students’ learning activities may aggravate their mental health.

From what is mentioned above, it can be seen that the pandemic outbreak has had a great impact on various aspects of graduate students’ lives. However, existing studies have mainly investigated elementary, middle, or undergraduate students (Currie et al., 2020; Moore and Lucas, 2020; Chen et al., 2021), few studies about the impact of the pandemic on graduate students’ course and social activities have been carried out yet, as well as investigations into the mental health of graduate students. It is essential to understand the impact of the pandemic on the learning process of graduate students so as to implement effective intervention and assist them in overcoming the difficulties. Therefore, a questionnaire was developed in this study to investigate the extent to which graduate students’ course activities and social activities were affected by the pandemic. In addition, previous studies mostly looked at students as a homogenous whole, ignoring the inter-group heterogeneity. Thus, we used the latent class analysis (LCA) method, a person-oriented method, to classify the degree to which graduate students’ learning activities affected by the pandemic and to explore the differences between different groups and their mental health status. In this way, we not only gained an understanding of the degree to which graduate students’ learning was affected by the pandemic and their psychological health with the background of pandemic prevention and control, but we also provide a reference for targeted psychological counseling.

## Materials and Methods

### Participants

A total of 2,818 graduate students were selected by a snowball sampling from 33 provinces or autonomous regions in China through the popular Chinese professional survey website *Wenjuanxing*.^1^ All respondents participated voluntarily, anonymously, and provided informed consent. This study was approved by the local Ethics Committee of the School of Psychology, South China Normal University (SCNU-PSY-2021-021). The group average age was 24.95 ± 3.31 years.

### Measures

#### The Pandemic’s Impact on Graduate Students Scale

The pandemic’s impact on graduate students scale was developed to measure the extent to which graduate students’ course and social activities were affected by the pandemic. The scale was comprised of two sections. Introductory section is related to course activities, including three items: “To what extent is your course arrangement affected by the pandemic?,” “To what extent is your assessment arrangement affected by the pandemic?,” “To what extent is your professional course learning affected by the pandemic?.” Section “Materials and Methods” connected to social activities, including three items: “To what extent is the academic exchange with your tutor affected by the pandemic?,” “To what extent is the academic exchange with your classmates affected by the pandemic?,” “To what extent is the general communication with your classmates affected by the pandemic?.” Respondents reported their answers using a four-item Likert rating scale: “not affected,” “slightly affected,” “moderately affected,” and “largely affected.” The score ranges from 1 to 4. The higher the score, the greater the impact. Results of the confirmatory factor analyses suggest that the model fit the data well: *χ*^2^(8) = 103.08, *p* < 0.01, RMSEA = 0.065, CFI = 0.99, TLI = 0.98, SRMR = 0.02. The Chinese version of the scale has a Cronbach’s alpha equal to 0.892.

#### Self-Rating Anxiety Scale

The self-rating anxiety scale was used to assess people’s anxiety in the past week (Zung, 1971). The scale included 20 self-evaluation items out of which five items were scored in the reverse order. The scale was a 4-item Likert scale with scores on responses ranging from 1 (occasionally) to 4 (always). The sum of the item scores was referred to as the total rough score, and the total rough score was multiplied by 1.25 to obtain the standard score. According to the Chinese norm, the anxiety scores were interpreted as follows: (50–59) scores indicated mild anxiety; (60–69) scores indicated moderate anxiety; and scores >69 indicated severe anxiety (Zhang and He, 1998). The Cronbach’s alpha coefficient for self-rating anxiety scale (SAS) in this study is 0.847.

#### Self-Rating Depression Scale

The self-rating depression scale was used to assess people’s depression in the past week (Zung, 1965). The scale included 20 self-evaluation items out of which 10 items were scored in the reverse order. The participants responded on the 4-item Likert scale with scores ranging from 1 (occasionally) to 4 (always). The total rough score was the sum of scores of all items, and the total rough score was multiplied by 1.25 to obtain the standard score. According to the Chinese norm, depression scores were interpreted as follows: (53–61) scores indicated mild depression; (62–71) scores indicated moderate depression; and scores >72 indicated severe depression (Wang et al., 1986). The Cronbach’s alpha coefficient for self-rating depression scale (SDS) in this study is 0.873.

#### Social Avoidance and Distress Scale

The social avoidance and distress scale was used to assess people’s tendency to avoid social interactions and feelings of distress (Watson and Friend, 1969). Avoidance is a behavioral response, and distress is an emotional response. The scale included 28 self-evaluation items, with 14 items assessing social avoidance and 14 items assessing anxiety. The scale was a two-point scoring scale, in which 1 stood for “yes” and 2 stood for “no.” The higher the sum of items score, the higher the degree of avoidance and distress. The Chinese scale used in this study was the revised version of the Rating Scales for Mental Health (Wang, 1993). The Cronbach’s alpha coefficient for social avoidance and distress scale (SAD) in this study is 0.920.

The three scales used in this study basically maintain their original versions, and only the sentences of a few items were revised according to the current context to ensure that the participants understood the meaning of each item.

### Statistical Analysis

Mplus 8.3 was used to conduct the LCA. Four steps were included in the data analysis process, as follows: first, the “not affected” and “slightly affected” options in the self-developed scale were recoded as 0. The “moderately affected” and “largely affected” options in the questionnaire were recoded as 1. The second step was to analyze the patterns of the graduate students affected by the pandemic using LCA. The third step was to build a multinomial logistic regression model based on the results of the second step, using the classification results of the LCA as dependent variables and the grade and major as the independent variables. The purpose was to understand the impacts of the grade and major on the classification. The fourth and last step was also based on the results of the second step, in which the classification results of the LCA were used as independent variables and the scores of anxiety, depression, social anxiety, and social avoidance scales were used as dependent variables. An analysis of variance was performed to understand the differences in mental health status in various classes.

In the LCA, the evaluation indicators for model fitting included as: (1) Akaike information criteria (AIC), Bayesian information criteria (BIC), and sample-adjusted BIC (aBIC). The smaller the above indexes values, the better the fit of the model and the greater the performance of the model; (2) Entropy statistics. The greater the entropy value, the higher the accuracy of the classification. At entropy = 0.8, the accuracy of the classification exceeded 90%; and (3) Lo–Mendell–Rubin likelihood ratio test (LMR), sample-adjusted LMR (aLMR), and bootstrap-based likelihood ratio test (BLRT). When the *p*-value reaches a significant level, it suggests that *k* classes are significantly better than the model of *k*−1 classes. All the statistical tests were two-tailed, and the test level was *α* = 0.05. The present study comprehensively considers the results of various indicators and combines them to determine the best model of graduate students’ learning activities. Harman’s single factor test was used to identify common method variance. The total variance extracted by one factor was 19.6%, which was less than 40%, indicating that there was no significant common method variance.

## Results

### Descriptive Statistics

The general demographic characteristics of the participants are shown in Table 1. The group average age was 24.95 ± 3.31 years. In total, 567 participants were male (20.12%) and 2,251 were female (79.88%). Table 2 shows the mental health status of graduate students. A 20.19% of graduate students suffered from anxiety, and 33.39% of them experienced different levels of depression.

**Table 1:** General demographic characteristics of respondents.

Demographic characteristic	Category	*N*	Percentage
Gender	Male	567	20.12
	Female	2,251	79.88
Grade	First-year graduate	1,674	59.40
	Second-year graduate	772	27.40
	Third-year graduate	372	13.20
Major	Humanities	439	15.58
	Science and engineering	394	13.98
	Social studies	1,985	70.44

**Table 2:** Descriptive analysis of mental health status.

Variable	Mild	Moderate	Severe	MD	SD
Anxiety	409 (14.51%)	121 (4.29%)	39 (1.38%)	42.47	9.67
Depression	615 (21.82%)	291 (10.33%)	35 (1.24%)	47.52	10.89
Social avoidance	–	–	–	6.48	3.91
Social anxiety	–	–	–	6.18	4.18
Social avoidance and distress	–	–	–	12.67	7.69

### Exploratory LCA

The number of classes was gradually increased from the initial 1-class model to a 6-class model. The goodness-of-fit indexes of all the models are presented in Table 3. The AIC decreased from the benchmark model to the 5-class model but started to rise in the 6-class model. Among the fitting results of the six models, the 4-class model had the lowest BIC and aBIC values. For LMR, aLMR, and BLRT, when the four classes were retained, these three indicators all reached significant levels. However, when five classes were retained, these values were no longer significant, indicating that the 4-class model was better than 3-class model, whereas the 5-class model had no obvious advantage over the 4-class model. After integrating the index results of each model, the 4-class model was selected as the optimal model.

**Table 3:** The goodness-of-fit indexes of exploratory LCA.

Model	AIC	BIC	aBIC	LMR	aLMR	BLRT	Entropy	Latent class probability
1	23,158.18	23,193.84	23,174.78	–	–	–	–	1.00
2	18,177.69	18,254.96	18,213.65	***	***	***	0.86	0.51/0.49
3	16,933.63	17,052.51	16,988.96	***	***	***	**0.89**	0.19/0.44/0.37
4	16,274.54	**16,435.02**	**16,349.23**	***	***	***	0.87	0.35/0.32/0.19/0.14
5	**16,267.47**	16,469.56	16,361.53	0.129	0.134	0.013	0.84	0.18/0.10/0.31/0.06/0.35
6	16,271.81	16,515.50	16,385.23	0.163	0.168	0.600	0.85	0.01/0.08/0.06/0.18/0.32/0.35

^***^p < 0.001. Bold type denotes the suggested best model by the respective index.

**Figure 1:**
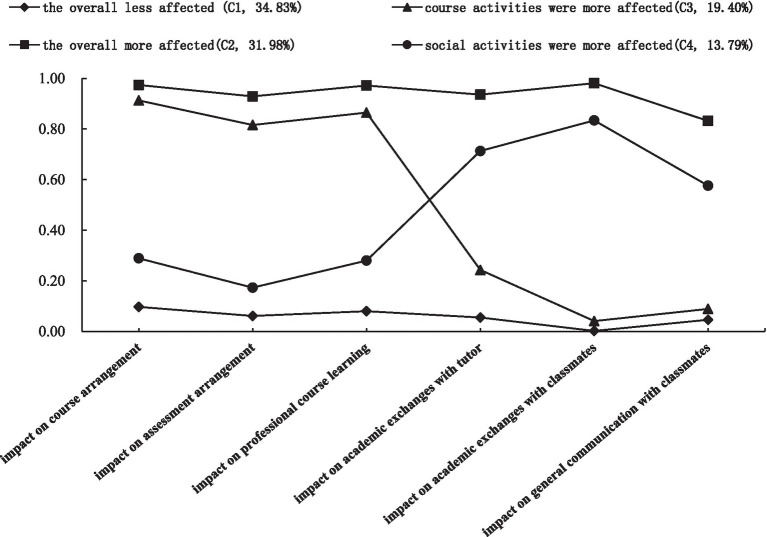
Conditional probability distribution diagram of each latent class.

The conditional probability of each latent class for each item was further explored and its distribution diagram is shown in Figure 1. The results revealed that class 1 (34.83%) and class 2 (31.97%) accounted for a relatively large proportion, whereas class 3 (19.40%) and class 4 (13.79%) accounted for a smaller proportion. Next, each class was named based on the patterns of responses. For all items, individuals in class 1 tended to report “not affected” and “slightly affected,” indicating they experienced greater impacts on both activities; thus, they were placed in “the overall less affected” class. On the contrary, out of six entries, the conditional probabilities of “moderately affected” and “largely affected” were over 0.8 for individuals in class 2, suggesting they were rarely affected neither in course activities nor social activities; thus, they were labeled as “the overall more affected.” Individuals in the third class had a higher tendency to answer “moderately affected” and “largely affected” for the first three questions, whereas in the last three questions, they had a lower tendency. Individuals in class 4 showed the opposite trend. The first three questions mainly reflected the pandemic’s impacts on course activities, whereas the latter three questions are mainly concerned with impacts on social activities. Therefore, individuals in classes 3 and 4 were named “course activities were more affected” and “social activities were more affected,” respectively.

### The Influence of Demographic Variables on Classification

Based on the results of classification, the impact of demographic variables on the classification was explored. Using the four classes as dependent variables and both grade and major as independent variables, a multinomial logistic regression analysis was performed. Taking class 1 “the overall less affected” as the benchmark reference class, the odd ratio coefficients (ORs) were obtained to reflect the effect sizes of different grades and majors on each latent class. The next step was recoding the categorical variables as dummy variables. When examining the grade effect, two dummy variables were set, with the first year of a Master’s degree as the baseline level. Similarly, when the effect of major was examined, two dummy variables were set with the humanities as the baseline level. The results of the multinomial logistic regression are presented in Table 4.

**Table 4:** Multinomial logistic regression of demographic variables on each latent class.

	The overall more affected		Course activities were more affected		Social activities were more affected	
	OR	CI (95%)	OR	CI (95%)	OR	CI (95%)
*Grade*						
Second-year graduate students	2.89^***^	2.22–3.56	1.87^**^	1.34–2.39	2.35^**^	1.57–3.13
Third-year graduate students	2.34^***^	1.66–3.03	1.13	0.69–1.57	2.16^*^	1.26–3.06
*Major*						
Science and engineering	0.80	0.51–1.08	0.79	0.45–1.14	1.88	0.85–2.91
Social studies	0.82	0.60–1.04	0.79	0.54–1.05	1.46	0.79–2.13

^*^p < 0.05, ^**^p < 0.01, and ^***^p < 0.001.

As shown in Table 4, grade is a significant predictor of the latent classes. To be more specific, compared with “the overall less affected” class, the proportions of postgraduates in the second-year (OR = 2.89, *p* < 0.001) and third-year (OR = 2.34, *p* < 0.001) in “the overall more affected” class were greater. In the “course activities were more affected” class, the odds for second-year postgraduates (OR = 1.87, *p* < 0.01) were higher than those of first-year and third-year postgraduates. For postgraduates in the “social activities were more affected” class, second-year (OR = 2.35, *p* < 0.01) and third-year students (OR = 2.16, *p* < 0.05) accounted for greater proportions than first-year students.

### The Mental Status of Graduate Students in Various Classes

The scores of graduate students’ anxiety, depression, social avoidance, social anxiety, and social avoidance distress scales in various classes are shown in Table 5, and the analysis of variance results is presented in Table 6. As shown in the two tables (Tables 5 and 6), there were significant differences in the scores for anxiety (*F* = 61.13, *p* < 0.001), depression (*F* = 67.61, *p* < 0.001), and social anxiety (*F* = 9.54, *p* < 0.05) among the four classes. In terms of anxiety scores, “the overall more affected” class (44.41 ± 0.35) was significantly higher than the “course activities were more affected” class (42.07 ± 0.43) and the “social activities were more affected” class (42.76 ± 0.56). “The overall less affected” (40.8 ± 0.31) had the lowest score. Similarly, the depression scores of “the overall more affected” class (49.95 ± 0.39) were significantly higher than that of the “course activities were more affected” (47.12 ± 0.50) and “social activities were more affected” (47.02 ± 0.65) classes. “The overall less affected” class (45.70 ± 0.35) had the lowest depression score. For social anxiety, the score of “the overall more affected” (6.52 ± 0.14) was significantly higher than those of the other three groups. There were no significant differences in the social anxiety scores among the “course activities were more affected” (6.04 ± 0.19), “social activities were more affected” (6.25 ± 0.26), and “the overall less affected” (5.93 ± 0.14) classes.

**Table 5:** Mental health scores of graduate students in various latent classes.

Variable	The overall less affected (C1)	The overall more affected (C2)	Course activities were more affected (C3)	Social activities were more affected (C4)
Anxiety	40.8 ± 0.31	44.41 ± 0.35	42.07 ± 0.43	42.76 ± 0.56
Depression	45.70 ± 0.35	49.95 ± 0.39	47.12 ± 0.50	47.02 ± 0.65
Social avoidance	6.42 ± 0.13	6.50 ± 0.13	6.43 ± 0.18	6.70 ± 0.24
Social anxiety	5.93 ± 0.14	6.52 ± 0.14	6.04 ± 0.19	6.25 ± 0.26
Social avoidance and distress	12.34 ± 0.26	13.02 ± 0.26	12.47 ± 0.36	12.95 ± 0.47

**Table 6:** Different tests of the mental health status of graduate students in various latent classes.

Variable	*F*	C1 vs. C2	C1 vs. C3	C1 vs. C4	C2 vs. C3	C2 vs. C4	C3 vs. C4	*Post hoc* comparison
Anxiety	61.13^***^	59.72^***^	5.39^*^	8.87^**^	16.93^***^	5.70^*^	0.92	C2 > C4 = C3 > C1
Depression	67.61^***^	66.55^***^	5.20^*^	2.98	19.51^***^	13.68^***^	0.02	C2 > C3 = C4 > C1
Social avoidance	1.17	0.22	0.00	1.03	0.11	0.47	0.79	–
Social anxiety	9.54^*^	8.65^**^	0.20	1.15	3.86	0.74	0.43	C2 > C4 = C3 = C1
Social avoidance and distress	4.12	3.38	0.07	1.21	1.50	0.01	0.65	–

^*^p < 0.05, ^**^p < 0.01, and ^***^p < 0.001.

## Discussion

### LCA of Graduate Students’ Learning Activities Affected by the Pandemic

An LCA was used to explore the extent to which graduate students’ course activities and social activities were affected by the pandemic. The results suggested that the 4-class model was the optimum. The four classes were “the overall less affected,” “the overall more affected,” and “course activities were more affected,” and “social activities were more affected,” respectively.

“The overall less affected” occupied the largest proportion, with 34.83% of the individuals belonging to the class. A possible reason for this class is that they were able to adjust their learning strategies in time to adapt to the changes in their surroundings (Blake et al., 2021). “The overall more affected” class was greatly affected in both course activities and social activities, accounting for 31.98% of the individuals. Quite a few graduate students had difficulty adapting to the changes in their course learning and communication modes (Blake et al., 2021). Based on the Triadic Reciprocal Determinism Theory, a person’s behavior both influences and is influenced by personal factors and the social environment (Bandura, 1978). During the pandemic, there are major and explicit changes in learning environment for students, resulting in less learning engagement among students (Wang and Cai, 2021). Graduate students in the “course activities were more affected” class were mainly affected in their course study, and 19.40% of individuals belonged to this class. During the quarantine, teaching methods changed from offline to online and the learning environment at home may be unsatisfactory (Kapasia et al., 2020; Rasheed et al., 2020); thus, some students experience major changes in course activities. The fourth class is the “social activities were more affected” class and 13.79% of postgraduates were classified in this class. During the pandemic period, the communications of some postgraduates with their supervisors and classmates were considerably affected because of barriers such as poor internet service, untimely feedback, and communication difficulties, and these problems make it challenging for students to proceed with their learning (Currie et al., 2020; Blake et al., 2021).

### Differences in the Effects of the Pandemic on Graduate Students in Different Grades

A multinomial logistic regression analysis was further used to explore the impact of demographic variables on classification. There were no significant differences in the degree to which graduate students of different majors were affected by the pandemic. In terms of course activities, the time investment of students in science and engineering, humanities, and social studies were not significantly different (Fan et al., 2020). In terms of social activities, students of different majors can easily communicate with teachers and classmates through online meetings, telephone calls, and WeChat in the era of rapid Internet development. Consequently, there were no significant differences in the impact of the pandemic on graduate students having different majors.

The learning activities of graduate students in different grades were affected to varying degrees. Specifically, compared with the “the overall less affected,” second-year and third-year master’s students accounted for a larger proportion of “the overall more affected” and “social activities were more affected” classes. This result may result from the following: On the one hand, the first half of 2020 was considered the most severe period of the domestic pandemic. At that time, the first-year graduate students had not yet enrolled. By the time freshmen enrolled in September, the teaching and learning activities of most universities, except for those in areas where the pandemic had recurred, had already returned to normal; consequently, the learning of the first-year students was affected less during the pandemic. On the other hand, compared with the first-year graduate students, senior students suffered from more pressure related to studies and scientific research, such as experimental design, data collection, and academic writing. They were more reliant on the experimental conditions and equipment provided by schools (Yi et al., 2020). Students who are about to graduate are also confronted with difficulties such as graduation theses, internships, employment, and further studies (Liu and He, 2020). Online learning, communication modes, and home quarantine, however, greatly inconvenienced their studies, leading to communication difficulties and low efficiency levels (Blake et al., 2021). Therefore, students in the second and third years were more likely to be affected by the pandemic. In the “course activities were more affected” class, second-year graduate students accounted for the highest proportion. In the second year, there are still many optional courses for graduate students to complete, and students may face problems with online courses, such as difficult teacher-student communication, unstable online teaching platforms, and teachers with insufficient online teaching experience (Zhang et al., 2020). These factors may greatly impact course activities. However, students in the third year have completed most professional courses, and their main tasks are to write a graduate thesis and conduct internships; consequently, their course activities were less affected.

Thus, in the context of the current pandemic, more attention should be paid to the learning activities of second-year and third-year students. For second-year graduate students, schools need to focus more on students’ learning curriculums, understand their demands, and provide them timely assistance. For third-year students, schools should create more options to release the pressure on finding an internship or employment. Additionally, their tutors could provide more guidance on their theses, providing additional support to help them overcome the crisis.

### Differences in the Mental Status of Graduate Students in Various Classes

Here, differences in the mental health of graduate students in various classes were explored using an analysis of variance. The levels of anxiety, depression, and social anxiety in graduate students of “the overall more affected” class were significantly higher than those of the other three groups. Obstacles in course learning, the process of experiment and social interaction likely cause anxiety and depression (Barrot et al., 2021). In addition, distance learning, as a new route of education, would raise anxiety levels (Adhikari et al., 2020). Furthermore, social distancing may also result in psychological consequences such as loss of motivation, loss of self-worth, anxiety, and depression (Ge et al., 2017; Mora-gallegos and Fornaguera, 2019; Williams et al., 2020). These factors mentioned above lead to serious psychological outcomes among some graduate students.

In comparison to “the overall more affected” class, students in the “course activities were more affected” class were less affected in their social activities. Social support exerts a considerably positive effect on individuals. It enables individuals to experience social rewards and enhances their subjective wellbeing, thereby promoting a positive mental state (Cohen, 2004; Leal Filho et al., 2021; Qiu et al., 2021). Individuals who have strong social support during the pandemic also maintain a low level of anxiety, depression, and stress (Cao et al., 2020). When their course activities were greatly affected, such graduate students actively engaged in academic exchanges with their tutors and peers through online methods, such as WeChat, telephone, and video to obtain help and support, positively promoting their learning effectiveness (Naddeo et al., 2021). Thus, their levels of anxiety, depression, and social anxiety were lower than students in “the overall more affected” class. Although graduate students in “social activities were more affected” were hindered to some extent in communication with their tutors and classmates, they were capable of adjusting to the changes in curriculum and examination arrangements during the pandemic (Blake et al., 2021). Some students also claimed that they enjoyed this new format of learning, which allowed them to arrange their time freely (Blake et al., 2021). Therefore, the anxiety, depression, and social anxiety levels they experienced were lower than those in “the overall more affected” class. Students in the “the overall less affected” class were able to adapt to various changes during the pandemic (Farias Bezerra et al., 2021); consequently, their scores were the lowest among the four groups on each scale, and they were all in good mental condition.

The current study showed that the levels of anxiety, depression, and social anxiety in individuals of “the overall more affected” class were significantly higher than those of the other three groups, and nearly one-third of graduate students belonged to this class. Schools and teachers should give more attention and care to these students. As a result of the pandemic, a number of graduate students have developed mental health problems, such as anxiety and depression. During the school year, schools and teachers play crucial roles in reducing student anxiety and depression (Jowsey et al., 2020). Therefore, with the background of the current normalization after the pandemic, schools and teachers cannot ignore student mental health, and targeted solutions to their problems should be provided for different types of graduate students. For all students, especially those in the “the overall more affected” class, universities should provide more psychological counseling to alleviate student anxiety and depression. For those whose course activities were greatly affected, given that students may lose motivation and goals in times of COVID-19 (Williams et al., 2020), it can also be helpful to offer career planning classes or lectures to help students set clearer goals and prepare ahead of time. Furthermore, since course activities are important parts of school lives, the teaching model is imperative to be optimized in times of COVID-19. For instance, to include more online interactive activities in class to help students actively engage in class, to segment teaching contents into a few parts to help students focus (Bao, 2020), and to provide online teaching training for teachers to ensure the teaching effects (Zhang et al., 2020). For those who were greatly affected by social activities, communications with tutors and fellow students, as well as any concerns they may have, are helpful in improving their mental health (Li et al., 2019; Wang et al., 2020). Accordingly, tutors should be more concerned not only with the students’ learning, but also with their living conditions and mental health, and provide them assistance if possible. For example, giving more assistance in assessment of student work such as graduation thesis. And video tutoring can be used to provide timely and prompt help for students. More importantly, tutors should ask and understand the mental status of students and avoid putting too much pressure on them. In this way, not only can students increase learning efficiency, but also reduce their anxiety (Dorrian and Wache, 2009; Jowsey et al., 2020).

## Strengths and Limitations

This study has some strengths and limitations. In this study, a large sample size (2,818 respondents) enables us to obtain robust analysis results while being able to obtain reliable trends and relationships between variables. Furthermore, graduate students all over the world were greatly affected by the pandemic, which has resulted in the development of mental health issues. They need advice and help to cope with these issues, and this study yields valuable insights regarding necessary interventions to help mitigate the impacts of mental health problems resulting from the pandemic.

Regarding the limitations, first, participants were sampled from one specific area, indicating that they were homogeneous to some extent. Future research should include more representative samples. Second, this was a cross-sectional study. A longitudinal study can be conducted in the future to explore the differences in student mental status during and after the pandemic. Third, self-report measures were used in this study, and social desirability bias may have affected the results.

## Conclusion

Four latent classes were identified with respect to the extent to which graduate students’ learning activities were impacted by the pandemic. The impacts on the learning activities of second-year and third-year graduate students were greater than on first-year graduate students. Due to the effects of the COVID-19 pandemic, a number of graduate students have developed mental problems, such as anxiety and depression. Notably, students in “the overall more affected” class suffer from higher levels of anxiety, depression, and social anxiety. Under the current backdrop of a new normal, schools and teachers should pay attention to student mental health, providing targeted assistance to different students, and this research provides valuable insights into the interventions necessary to help mitigate the effects of mental health issues caused by the pandemic.

## Data Availability Statement

The raw data supporting the conclusions of this article will be made available by the authors, without undue reservation.

## Ethics Statement

The studies involving human participants were reviewed and approved by Ethics Committee of the School of Psychology, South China Normal University (SCNU-PSY-2021-021). The patients/participants provided their written informed consent to participate in this study.

## Author Contributions

JW contributed to the data analysis and the interpretation of the results. JW and DK drafted the first version. ZL and MZ revised the drafts of the manuscript. All authors contributed to the conception and design of the paper and the data collection, and have read and approved this version.

## Funding

This research was funded by the key project of the Chinese National Social Science Fund, “Research on Problems and System Innovation in Educational Integrated Development in Guangdong-Hong Kong-Macao Greater Bay Area” (grant number: AGA200016).

## Conflict of Interest

The authors declare that the research was conducted in the absence of any commercial or financial relationships that could be construed as a potential conflict of interest.

## Publisher’s Note

All claims expressed in this article are solely those of the authors and do not necessarily represent those of their affiliated organizations, or those of the publisher, the editors and the reviewers. Any product that may be evaluated in this article, or claim that may be made by its manufacturer, is not guaranteed or endorsed by the publisher.

## Footnotes

^1^ www.sojump.com
